# Identifying seasonal and temporal trends in the pressures experienced by hospitals related to unscheduled care

**DOI:** 10.1186/s12913-016-1555-7

**Published:** 2016-07-26

**Authors:** NJ Walker, HC Van Woerden, V Kiparoglou, Y Yang

**Affiliations:** 1 NIHR Oxford Biomedical Research Centre, Churchill Hospital, Old Road, Headington, Oxford, OX3 7LE UK; 2 Institute of Primary Care & Public Health, Cardiff University, Cardiff, UK; 3 Centre for Health Science, University of the Highlands and Islands, Inverness, IV2 3JH UK; 4 Nuffield Department of Primary Care Health Science, University of Oxford, Oxford, UK

**Keywords:** Unscheduled care, Escalation scores, Hospital pressure, Emergency admissions, Annual cycles, Sines and cosines, Day of the week effect

## Abstract

**Background:**

As part of an electronic dashboard operated by Public Health Wales, senior managers at hospitals in Wales report daily “escalation” scores which reflect management opinion on the pressure a hospital is experiencing and ability to meet ongoing demand with respect to unscheduled care. An analysis was undertaken of escalation scores returned for 18 hospitals in Wales between the years 2006 and 2014 inclusive, with a view to identifying systematic temporal patterns in pressure experienced by hospitals in relation to unscheduled care.

**Methods:**

Exploratory data analysis indicated the presence of within-year cyclicity in average daily scores over all hospitals. In order to quantify this cyclicity, a Generalised Linear Mixed Model was fitted which incorporated a trigonometric function (sine and cosine) to capture within-year change in escalation. In addition, a 7-level categorical day of the week effect was fitted as well as a 3-level categorical Christmas holiday variable based on patterns observed in exploration of the raw data.

**Results:**

All of the main effects investigated were found to be statistically significant. Firstly, significant differences emerged in terms of overall pressure reported by individual hospitals. Furthermore, escalation scores were found to vary systematically within-year in a wave-like fashion for all hospitals (but not between hospitals) with the period of highest pressure consistently observed to occur in winter and lowest pressure in summer. In addition to this annual variation, pressure reported by hospitals was also found to be influenced by day of the week (low at weekends, high early in the working week) and especially low over the Christmas period but high immediately afterwards.

**Conclusions:**

Whilst unpredictable to a degree, quantifiable pressure experienced by hospitals can be anticipated according to models incorporating systematic temporal patterns. In the context of finite resources for healthcare services, these findings could optimise staffing schedules and inform resource utilisation.

## Background

Hospitals may struggle to operate effectively and meet quality standards when admission rates are high in relation to available resources such as beds, staff and operational capacity [1, 2]. Whilst healthcare planners use different strategies to control demand for elective treatments (for example by introducing a waiting list for treatments or operations, by bringing forward discharge to community or social care, and by ensuring resources match demand as far as possible), short term demand for emergency care is harder to manage. As most hospitals provide both elective and emergency care and run at high occupancy rates, there are times when hospitals may still struggle to fully meet demand and provide care for the population they serve. Although undesirable, such episodes are inevitable in light of finite resource allocation to healthcare budgets and the high operational costs involved in running a health service. Inadequate staffing and high bed occupancy rates result in overcrowding which can have an adverse influence on a range of patient outcomes [3] with some studies indicating a link to an increase in in-hospital patient mortality [4, 5] in addition to the spread of infectious pathogens within the hospital environment such as Methicillin-resistant *Staphylococcus Aureus* (MRSA) [6]. Overcrowding in hospitals is also observed to have an adverse effect on staff wellbeing [7]. The challenges faced in meeting the demands of running healthcare systems are likely to intensify in future as technologically complex and costly interventions increase in number and the demographic structure of target populations changes, particularly in terms of an increase in life expectancy and an elderly population with extensive comorbidity [8, 9].

Part of this pressure arises from unscheduled admissions referred to Emergency Departments (ED) and primary care practitioners due to sudden onset of illness or injury [10]. This is an especially challenging area of care to plan for due to its inherent unpredictability. In 2012–13, there were 5.3 million emergency admissions to hospitals in the UK, an increase of 47 % over the last 15 years [11] and overcrowding in ED has become a major problem [12].

There are nonetheless likely to be predictable temporal patterns in unscheduled care pressure, notably in relation to annual, weekly and daily cycles. Consistent monthly differences have been reported elsewhere in relation to objective measures of pressure such as the number of admissions and inpatient days [13]. Fullerton and Crawford [14] found that bed occupancy was influenced by both season and day of the week in relation to data from a large teaching hospital although these trends were found to vary between specialties. Furthermore, Jones [15] demonstrated the existence of long-term patterns in bed occupancy and highlighted the importance of identifying periods of time where this phenomenon is likely to be volatile. Understanding these patterns can help inform the planning of staff rotas and resource allocation.

In Wales, the pressure experienced by individual hospitals associated with unscheduled care is assessed and recorded as a so-called “escalation score”, a system first introduced in 2006 [16]. The score is reported according to four escalation status levels, which are used by all Health Boards in Wales and by the Welsh Ambulance Service NHS Trust. The levels are based on a number of triggers which define escalation to the next level. This system is designed to determine the appropriate response to escalating emergency pressures and “the actions necessary to protect core services in order to supply the best possible level of service with the resources available”. The scores are recorded on a “live” National Unscheduled Care Dashboard and are used to inform weekly unscheduled-care meetings of Health Board chief executives. Scores are determined by each locality based on Welsh Government guidelines, with a set of criteria defining the circumstances under which the escalation score should be raised to the next level and the actions that should be taken to bring the score down. The escalation score is recorded on a graded scale between 1 and 4 inclusive with only integer values used. Scores indicate the following states:

1. Steady state (hospital able to cope with current rate of admissions with available resources)
2. Moderate pressure (admissions likely to exceed capacity)
3. Severe pressure (admissions are exceeding capacity)
4. Extreme pressure (admissions significantly exceeding capacity)

A more detailed rationale is provided by Piggott et al. [16] including the triggers (based on, for example, the time taken for patients’ first contact with an assessing clinician) which underlie transition between the four states.

In the current paper, the authors analyse daily escalation scores from Welsh hospitals, where available, between the years 2006 and 2014 inclusive in order to gain an insight into temporal trends in unscheduled care pressure. This contrasts with other documented analyses of seasonal trends which utilise specific quantitative measures of hospital pressure such as bed occupancy [14], admission rates and in-hospital mortality [5]. Furthermore, we consider data from a number of hospitals simultaneously, affording an insight into possible heterogeneity between hospitals in terms of pressure intensity and temporal patterns. Computer models have an increasingly important part to play in optimising the management and delivery of emergency services [17–20]. Detailed information on systematic temporal patterns of pressure in hospitals is likely to enhance the accuracy and usefulness of such models. The models used here are relatively easy to implement and the results can usefully contribute to computer models as well as potentially informing staffing rosters in a climate where resource allocation needs to be optimised.

## Methods

Historic escalation scores from 1st January 2006 to 20 October 2014 were obtained for 18 major hospitals in Wales that provide unscheduled care. The dataset was provided by the NHS Wales Informatics Service, on behalf of the Unscheduled Care Lead for Wales. The time of day recorded in the database was not utilised in analysis, as there was uncertainty as to whether the time of day that each escalation score was electronically recorded reflected the time of day when the score was reported. To preserve anonymity, the hospitals are labelled with a numeric code (1–18) for presentational purposes.

In order to address potential issues regarding pseudo-replication (i.e. multiple observations per day), it was decided to use only one score per day for each hospital for analytical purposes. To this end, where more than one score was present, the median value on a given day was taken and rounded to the nearest integer where decimals were involved (e.g. 2.3 to 2, 1.5 to 2). This had the effect of returning a single score for each hospital on each day (where available) which was on the original integer scale between 1 and 4. Where scores had been stepped up or down during the day, the upward rounding of .5 favoured the higher of two values. This was felt to be the best reflection of the pressure the hospital had been under that day.

### Statistical model

The means of these median daily scores, computed as described above, were then calculated across all 18 hospitals with respect to (i) each date for which records were available spanning the period 2006 to 2014 inclusive (ii) calendar date within a year. These were inspected graphically in order to ascertain the presence of any systematic trends. In a similar way, the mean score was calculated for each day of the week across all hospitals in the dataset and these are presented in tabular form.

Statistical analysis was carried out in order to assess the possible existence of correlation between various temporal factors and the daily escalation scores and to define the nature of such relationships. A model was fitted in which the graded escalation score was modelled as a binary phenomenon where *n* = 3, such that scores 1–4 map to a number of positive outcomes 0–3 out of a maximum of 3. In order to assess the trends apparent from exploratory analysis, daily escalation scores were regressed against hospital, calendar date and day of the week.

### Trigonometric functions for annual cyclicity

**Fig. 1:**
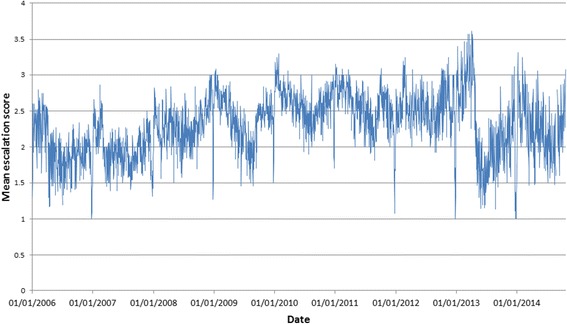
Change in mean escalation score averaged across all hospitals in Wales (2006–2014)

**Fig. 2:**
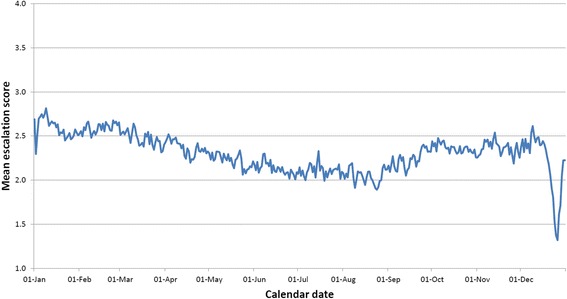
Mean daily escalation score calculated over all years in the dataset (2006–2014) and all 18 hospitals in the dataset for each calendar date within a year

Exploratory data analysis of the change in pressure by calendar date indicated the presence of within-year cyclicity in this phenomenon (Figs. 1 and 2). On this basis, it was decided to model within-year change in pressure using a trigonometric model incorporating both the sine and the cosine of the angle equivalent to calendar date (referred to from here on as θ). If we take January 1 as an arbitrary origin taking the value 0^0^, angles 90^0^, 180^0^ and 270^0^ approximate the passing of 3, 6 and 9 months respectively. A similar approach can be used to model any process that changes periodically in a wave-like motion over regular time cycles, e.g. changing temperature over a 24-h period, or total daily hours of sunlight recorded over a year.

Fitting both the sine and the cosine of angle θ allows for estimation of both the *amplitude* and *shift* of the underlying trend. According to mathematical convention, angle θ was measured in radians (2π radians = 360^0^). For more details on this modelling approach, see Stolwijk et al. [21].

### Day of the week and christmas effects

Day of the week was fitted as a categorical variable with 7 levels, one for each day. Exploratory data analysis indicated clear differences between days in recorded escalation, but not according to a clear parametric form, thus this less parsimonious approach fitting 7 independent effects was adopted.

Exploratory analyses also highlighted a lull in reported pressure over the Christmas period followed by a compensatory increase in the days immediately after the Christmas holiday. Thus a categorical variable “Christmas effect” was created which took a value of 2 for calendar dates December 21–28 inclusive, a value of 3 for dates January 3–9 inclusive and a baseline value of 1 for all other dates. These dates were chosen to approximate the periods for which these effects appeared to be present from looking at the data. Potential effects associated with other public holidays were not included in these models, although work elsewhere [22] suggests the possible existence of such effects.

### Statistical model

The response (escalation score) was modelled as a binomial process where *n* = 3 (thus four possible outcomes–0, 1, 2 and 3 corresponding to the four graded escalation scores 1, 2, 3 and 4 respectively). This was analysed using logistic regression in a Generalised Linear Model (GLM) framework, with a binomial distribution chosen to model the error in the response and a logit link function [23]. Escalation score was regressed against the following explanatory variables: hospital, sine and cosine of angle θ, day of the week and Christmas effect. In addition, the interactions between (i) hospital and sine (ii) hospital and cosine were included to assess whether the trigonometric model was different across hospitals. This model is summarised in the following equation:

$$ \mathrm{Logit}(P)=\alpha +{\beta}_{hosp}+\left({\beta}_{sine}+{\beta}_{hosp.sine}\right){x}_1+\left({\beta}_{cosine}+{\beta}_{hosp.cosine}\right){x}_2+{\beta}_{day}+{\beta}_{chr} $$

Where, $\mathrm{logit}(P)= \log \left(\frac{P}{1{\textstyle \mathit{\hbox{-}}}P}\right)$, *P* = binomial probability parameter, α = global constant, β_*hos****p***_ = hospital–specific constant, β_*sine*_ = global sine coefficient, β_*cosine*_ = global cosine coefficient, β_*hosp.sine*_ = hospital-specific sine coefficient, β_*hosp.cosine*_ = hospital-specific cosine coefficient, β_*day*_ = day of the week effect, β_*chr*_ = Christmas effect, *x*_1_ = sine of θ, *x*_2_ = cosine of θ.

Main effects associated with the interactions fitted (i.e. hospital, sine and cosine) were automatically retained where the relevant interactions were significant.

Binomial parameter *P* determines the expected escalation score for a given hospital on a given day. The predicted score can be estimated (returning a number on a continuum between 1 and 4) by back-transforming as follows:

$$ \widehat{E}=1+\left(3\times \left[\left( \exp \left(\widehat{B}\right)\right)/\left(\left(1+ \exp \left(\widehat{B}\right)\right)\right)\right]\right) $$

Where

*Ê* = expected escalation score and $\widehat{B}=\mathrm{logit}(P)$ calculated using the model estimates of the β parameters (β_*hosp*_, β_*sine*_ etc.).

A value of 1 is added to transform the binomial score (0–3) back to the scale of the escalation score (1–4).

Probabilities for each of the four possible escalation outcomes can be calculated as follows:

$$ \mathrm{Prob}(y)=\left(\begin{array}{c}3\\ {}y-1\end{array}\right){\widehat{P}}^{y-1}{\left(1-\widehat{P}\right)}^{3-y+1} $$

Where:

*y* = escalation score (1, 2, 3 or 4), $$ \left(\begin{array}{l}n\\ {}x\end{array}\right)=\frac{n!}{x!\left(n-x\right)!} $$ and $\widehat{P}=\frac{ \exp \left(\widehat{B}\right)}{\left(1+ \exp \left(\widehat{B}\right)\right)}$.

A degree of temporal autocorrelation in escalation score was anticipated further to the effect of model covariates such that an escalation score reported on one day has a high probability of being the same on the following day. Thus the above model was fitted using the Generalised Linear Mixed Model (GLMM) procedure in GenStat [24] incorporating day, nested within hospital, as a structured random effect. A first-order autoregressive structure (AR1) was selected to model this correlation. The statistical significance of putative autocorrelation was assessed by comparing deviance for this model against a similar model where autocorrelation was absent. Inference was based on estimated dispersion as opposed to a fixed value of 1, giving rise to approximate F-tests. The degrees of freedom for the denominator in relation to these tests were estimated according to the method presented by Kenward and Roger [25]. The model was fitted using the marginal method of Breslow and Clayton [26]. Model coefficients are reported on the logit scale.

The predicted escalation score *Ê* was calculated using the above formula separately for each hospital for each day of the year, matching calendar date to angle θ and averaging over other variables in the model. The predicted escalation profiles were then plotted and compared graphically with mean escalation scores by calendar date for the whole dataset.

Residuals from the best fitting model were assessed in terms of (i) the standardised deviance residual histogram (ii) standardised deviance residuals plotted against fitted values (iii) the normal-plot of standardised deviance residuals (iv) the half-normal plot of the same [24]. None of the above residuals highlighted significant deviation from model assumptions. All statistical analyses were carried out in GenStat 18th Edition [24].

## Results

Over the duration of the period investigated (1 Jan 2006 to 20 October 2014 inclusive) the number of escalation scores recorded by each hospital per day varied. Typically, one score only was available (*n* = 1 on 55.5 % of days) but sometimes this number was higher (*n* = 2 on 12.1 %; *n* = 3 on 5.9 % and *n* = 4 or more on 2.8 % of days) and sometimes no score was recorded at all (23.7 % of days). Days where no score was available were treated as missing data. The highest frequency of scores reported by a hospital on one day was 10. As described in the methods section, a single median value was taken on days on which more than one score was recorded.

**Fig. 3:**
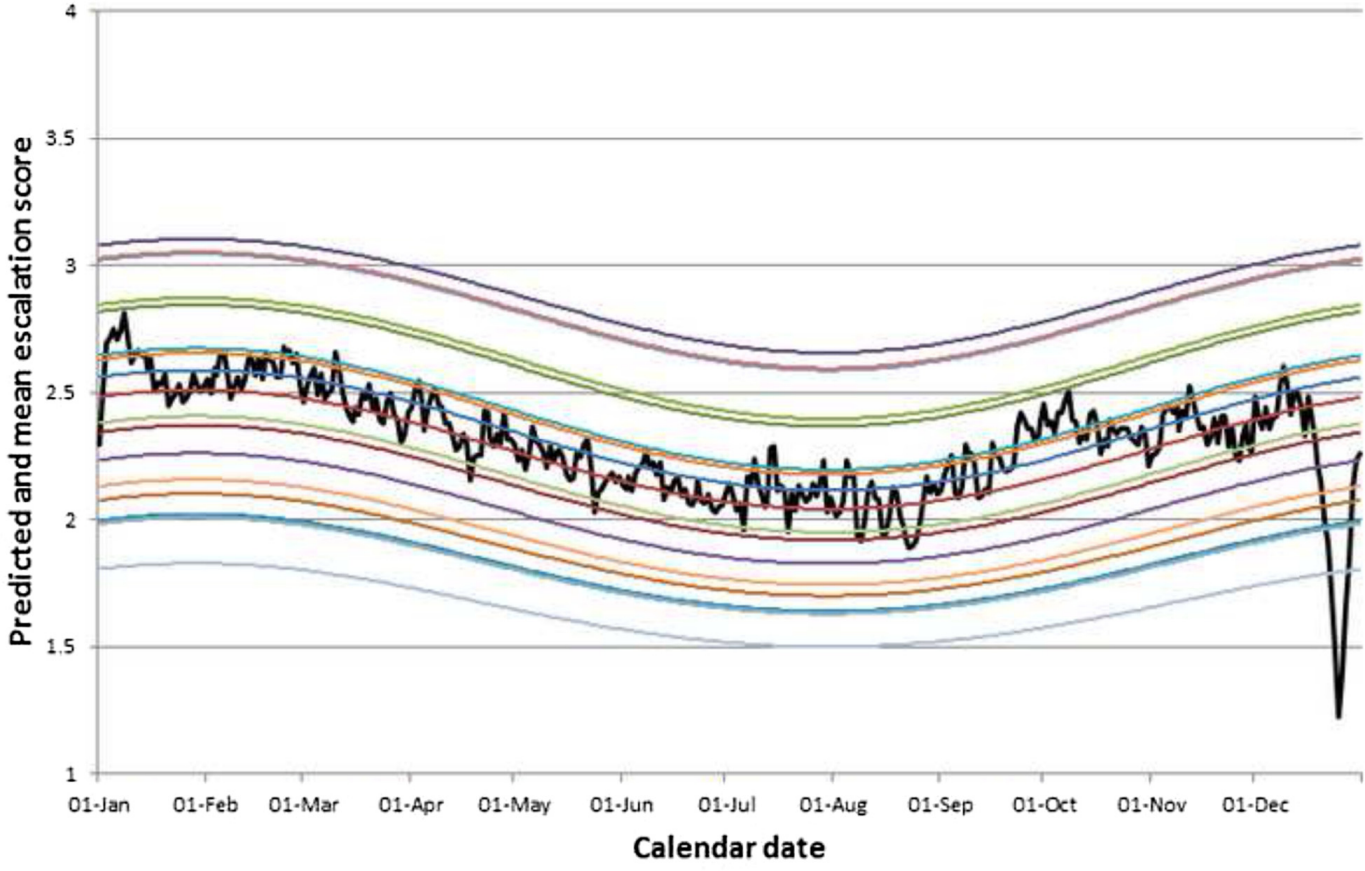
Predicted escalation scores (transformed from logit-scale model parameters) for the 18 different hospitals in the dataset by calendar date. Raw data averaged across all 18 hospitals denoted by black line

Clear differences in average escalation scores between hospitals and between days of the week can be seen from Table 1, supporting the inclusion of these effects in statistical models. Observation frequencies presented in Table 1 also highlight differing recording rates both between hospitals and between different days of the week with lower rates of reporting on weekend days. Furthermore, average escalation scores can be seen to cycle throughout the year when we consider average daily scores in terms of both the full timespan of data and within-year change only (Figs. 1 and 2). The reduction in escalation scores around the Christmas period is also evident from Figs. 1 and 2 (and to a lesser extent the corresponding spike afterwards). The trigonometric and hospital coefficients were used to calculate expected escalation (*Ê*) for each of the 18 hospitals and these are plotted alongside the overall average escalation score for each calendar date across the whole dataset (Fig. 3).

**Table 1:** Means of daily escalation scores for (i) hospitals (ii) days of the week between 2006-2014

Hospital	Number of days where data recorded (% of total days)	Mean of daily escalation score
1	2330 (72.5 %)	2.21
2	3051 (94.9 %)	2.60
3	2870 (89.3 %)	2.87
4	1315 (40.9 %)	1.87
5	2731 (89.5 %)	1.88
6	2562 (79.7 %)	2.31
7	3055 (95.0 %)	2.27
8	2557 (79.5 %)	2.66
9	1998 (62.1 %)	2.03
10	2184 (67.9 %)	2.42
11	2975 (92.5 %)	2.41
12	2855 (88.8 %)	2.81
13	2853 (88.7 %)	2.81
14	2969 (92.3 %)	2.18
15	2640 (82.1 %)	1.76
16	2739 (85.2 %)	1.82
17	1896 (59.0 %)	1.97
18	564 (17.5 %)	1.66
Day of the Week		
Sunday	5487 (66.3 %)	2.30
Monday	6710 (81.0 %)	2.46
Tuesday	6707 (81.2 %)	2.43
Wednesday	6643 (80.4 %)	2.35
Thursday	6647 (80.5 %)	2.30
Friday	6595 (79.8 %)	2.16
Saturday	5360 (64.9 %)	2.11

The deviance for the fully fitted GLMM including autocorrelation was 20901.9 as compared to a deviance of 50390.9 without. This equates to a reduction in deviance of 29489.0 for 1 extra degree of freedom (*p* < 0.001) for the fitting of AR1 parameter ϕ whose estimate and standard error were as follows: ϕ = 0.93, S.E. (ϕ) = 0.0018. Given the improvement in model fit described above, inference was based on the random-effect model including hospital/day autocorrelation. All main effects were found to be statistically significant, but both the trigonometric interactions were non-significant (for sine*hospital; F-statistic = 0.94, adjusted degrees of freedom = 17, 1301.6, *p* = 0.5 and for cosine*hospital; F-statistic = 1.3, adjusted d.f. = 17, 1304.7, *p* = 0.2) so the interactions were removed and inference is based on the model including main effects only (Table 2). The significance of both the sine and cosine of θ indicates that reported pressure follows a wave-like pattern within yearly cycles and that this phenomenon is observed across hospitals. It could be hypothesised that the nature of this pattern is different between hospitals but this is not supported by the results in relation to the interactions (in an earlier specification of the model not including autocorrelation, these interactions were significant, but the inclusion of AR1 correlation resulted in these effects being overridden).

**Table 2:** Coefficients (logit-scale), test statistics and p-values from full model, regressing escalation score against (i) sine of θ (ii) cosine of θ (iii) Day of the Week (iv) Christmas effect (v) Hospital

Variable	Level	β (logit scale)	F-stat (adjusted d.f.) & *p*-value
A		−0.57	
Sine		+0.15	29.2 (1, 1356.1), *p* < 0.001
Cosine		+0.28	97.6 (1, 1377.0), *p* < 0.001
Day of the week			591.4 (6, 34417.3), *p* < 0.001
	Sunday	0	
	Monday	+0.33	
	Tuesday	+0.28	
	Wednesday	+0.17	
	Thursday	+0.094	
	Friday	−0.11	
	Saturday	−0.29	
Christmas effect			221.4 (2, 20842.0), *p* < 0.001
	Baseline	0	
	Christmas	−0.80	
	Post-Christmas	+0.34	
Hospital			37.7 (17, 1200), *p* < 0.001
	Hospital 1	0	
	Hospital 2	+0.64	
	Hospital 3	+1.03	
	Hospital 4	−0.49	
	Hospital 5	−0.37	
	Hospital 6	+0.29	
	Hospital 7	+0.19	
	Hospital 8	+0.68	
	Hospital 9	−0.15	
	Hospital 10	+0.40	
	Hospital 11	+0.38	
	Hospital 12	+0.94	
	Hospital 13	+0.94	
	Hospital 14	+0.049	
	Hospital 15	−0.50	
	Hospital 16	−0.51	
	Hospital 17	−0.29	
	Hospital 18	−0.79	

Using calculus arguments and the sine/cosine model coefficients from the model (Table 2) allows for the estimation of predicted dates of lowest/highest pressure. According to model estimates the period of maximum pressure typically occurs in winter (28 January) and minimum pressure in summer (29 July) after adjusting for other model effects. In the absence of hospital*sine/cosine interactions, the dates of minimum/maximum pressure were assumed to be the same for all hospitals. The difference between minimum and maximum projected escalation varied from a lowest value of 0.33 (1.5 to 1.83) for hospital 18 to a highest value of 0.48 (2.20 to 2.67) for hospital 10. Both day of the week and Christmas effects were found to be statistically significant (day of the week – F-statistic = 591.4, adjusted d.f. = 6, 34417.3; *p* < 0.001; for Christmas effect–F-statistic = 221.4, adjusted d.f. = 2, 20842.0; *p* < 0.001). These results bear out the presence of reduced pressure attributable to unscheduled care up to and during the main days of Christmas (β = −0.80) and a modest increase after the Christmas period (β = +0.34). In terms of days of the week, the lowest escalation scores tend to be seen on Saturdays (β = −0.29) and the highest on Mondays (β = +0.33) relative to a baseline of 0 (Sunday).

## Discussion

In this paper, we present results from statistical models linking reported pressure scores to individual hospitals and (i) annual cycles (ii) day of the week (iii) the days during and after the Christmas period. Before considering these temporal patterns, it is clear that differences exist between hospitals in terms of overall pressure. This is evident from the raw data as per numbers presented in Table 1 and supported by the model results presented in Table 2. It may be significant that the three hospitals reporting the highest overall levels of pressure (hospitals 3, 12 and 13 in order of highest to lowest) were all based in major urban centres whereas the three hospitals reporting the lowest pressure (18, 15 and 16 in order of lowest to highest) were relatively rural.

The same general pattern of high and low pressure in winter and summer respectively is observed across hospitals. A significant part of the pressure in winter is likely to be associated with seasonal illness [27], although this phenomenon appears to be more general, being seen in apparently unrelated specialties such as orthopaedics [14]. It is possible to speculate that the reduced pressure from unscheduled care in summer may in part be due to a significant proportion of the population being away on holiday at any one time, although this is likely to be compensated for by a rise in the transient population during the tourist season.

As well as a difference in overall intensity of reported pressure, there is also some variability in the scale of difference between projected “quiet” and “busy” times of the year between hospitals. In the absence of hospital*sine/cosine interactions, this can be seen as a result of greater variation at hospitals with average scores close to an overall mean of 2.5 (i.e. halfway between the minimum score of 1 and maximum of 4) and least variation where average scores tend toward the extremes of 1 or 4. Such heterogeneity may in part reflect cultural differences in utilising escalation scores, although one can only speculate without extraneous information on metrics relating to the triggers underlying movement between escalation levels.

We see consistent differences in escalation according to day of the week, both with respect to raw data and model results. In particular, reported pressure tends to be lower at the weekend and then higher early in the working week (Monday and Tuesday). This finding is in broad agreement with results from analysis of bed occupancy rates which were found to be lower on average at the weekend [14, 22]. With respect to the data here, this may at first appear surprising if we anticipate a rise in unscheduled admissions on Friday and Saturday evenings due to alcohol related incidents. However, it may be the case that reduced discharge from hospital at weekends [28], combined with elective admissions on Mondays delays the adverse impact of weekend admissions on bed capacity and hence escalation score. There is some evidence to suggest that the risk of mortality may vary across the week [5, 29, 30]. This could be related to differences in staffing patterns, utilisation of hospital resources or differences in the profile of admissions [31]. Although within-day patterns were not considered in our analyses, there is likely to be systematic variation on this timescale too. For example, patterns in elective admissions and discharges have been observed to vary throughout the day in an analysis of data from a trust in England [32]. Circadian differences in admission time have also been reported in more specific instances such as asthma incidence [33]. However, it was not possible with the dataset in its present guise to investigate this aspect of temporal change further.

A phenomenon of delayed admission may be responsible for the sharp drop in reported pressure over the Christmas period, particularly given the subsequent rise observed in early January. This phenomenon may also be a function of increased discharge rate in the period prior to Christmas [32] which can have the effect of temporarily freeing up bed space.

Results from the current analysis broadly corroborate findings elsewhere with respect to seasonal change [14, 27], day of the week differences [22] and an effect associated with the Christmas period [14, 22]. However, the current model was predicated on a partially subjective measure taking values 1,2 3 or 4 as opposed to a quantitative metric (e.g. bed occupancy) taking continuous values over a wide range which is generally how this work has been approached elsewhere. The escalation score is a “catch-all” measure for a hospital which is influenced by a number of phenomena such as seasonal illness, staffing levels and different specialties which are themselves subject to particular patterns in pressure over time [14]. An advantage of working with this dataset was the ability to look at individual hospitals and the similarities/differences between them allowing us to assess whether one model is appropriate for all hospitals or not.

Whilst the best-fitting model provides statistical evidence of systematic temporal patterns in pressure arising from unscheduled care, the presence of significant autocorrelation in hospitals over time indicates a strong association between reported escalation on a given day and the day after. This aspect of pressure cannot be anticipated on the basis of long-term temporal patterns. However, the finding does imply that when pressure reaches a critical level, it is likely to remain there for a number of days.

Caution must be attached to the interpretation of these results in light of the subjective nature of the outcome (reported escalation). Differences in model results between hospitals may be due to differing escalation score thresholds among the respective hospital managers. This highlights a potential advantage of moving to a more objective measure for capturing pressure attributable to unscheduled care based on recorded metrics (e.g. percentage bed occupancy or hours lost by waiting ambulances) and work on the development of such a measure using data from the Welsh dashboard is in progress.

The trigonometric models used, whilst highlighting significant seasonal trends in hospital-level pressure (evident from the raw data as per Fig. 2) are nonetheless restrictive with respect to enforcing a half-year gap between the maximum and minimum point in the cycle. It may be that this gap is uneven (e.g. 5 & 7 months) and/or that this varies between hospitals. The extent of the data allows for potentially more sophisticated modelling such as time-series analysis which has been used, for example, in forecasting emergency call rates [34] although this would be facilitated by the existence of a measure of hospital pressure on a continuous scale (which can thus be modelled with Gaussian error) as opposed to the discrete measure used here.

## Conclusions

An obvious solution to overcrowding in EDs is to increase nursing staff numbers above a critical level at all times but in the context of increasing healthcare costs and limits on spending more pragmatic solutions need to be sought [35]. A number of studies have looked into the identification of an optimal nurse to patient ratio [36, 37] although no consistent picture emerges as to what this figure might be. It is evident that greater nursing hours per patient are associated with better patient outcomes [5, 38, 39] although there are also dangers attached to overuse of individual staff, both for nurse and patient. Whilst it may not be feasible in the current climate to simply assign staff numbers in excess of a pre-defined threshold, models taking into account the temporal patterns we have considered here may assist in adjusting staff rosters to optimise service in the context of limited resources. There is an increasing need for sophisticated evidence-based modelling to ensure cost-correct staffing [40] and detailed quantitative information on systematic temporal trends can make an important contribution in this endeavour.

Although the data were recorded in Wales it is likely that the findings with respect to seasonal cycles, day of the week differences and a rise and fall during and after the Christmas period are likely to be generalisable to other parts of the UK. However, we have also seen within this dataset that a degree of inter-hospital difference may be present (certainly in terms of overall pressure, possibly also in terms of within-year patterns in change), thus it may be preferable for hospitals to be considered individually in this regard. Generalisations to systems outside the UK are more difficult to make, but some commonality may be expected in culturally similar countries (e.g. in Europe, USA) with geographical proximity to the UK also likely to have some bearing.

## Abbreviations

ED, Emergency Department; GLM, Generalised Linear Model; GLMM, Generalised Linear Mixed Model; MRSA, Methicillin-resistant Staphylococcus Aureus
